# Investigating the impact of vitrification on bovine ovarian tissue morphology, follicle survival, and transcriptomic signature

**DOI:** 10.1007/s10815-024-03038-4

**Published:** 2024-02-15

**Authors:** Spyridon P. Deligiannis, Keiu Kask, Vijayachitra Modhukur, Nina Boskovic, Marilin Ivask, Ülle Jaakma, Pauliina Damdimopoulou, Timo Tuuri, Agne Velthut-Meikas, Andres Salumets

**Affiliations:** 1 Division of Obstetrics and Gynecology, Department of Clinical Science, Intervention and Technology, Karolinska Institutet, Huddinge, 14186 Stockholm Sweden; 2 Department of Gynecology and Reproductive Medicine, Karolinska University Hospital, 14186 Stockholm, Sweden; 3 Department of Obstetrics and Gynaecology, Institute of Clinical Medicine, University of Tartu, 50406 Tartu, Estonia; 4 Department of Obstetrics and Gynecology, University of Helsinki, 00290 Helsinki, Finland; 5 Competence Centre of Health Technologies, 50411 Tartu, Estonia; 6 Department of Biosciences and Nutrition, Karolinska Institutet, 14183 Huddinge, Sweden; 7 Department of Pathophysiology, Institute of Biomedicine and Translational Medicine, University of Tartu, 50411 Tartu, Estonia; 8 Institute of Veterinary Medicine and Animal Sciences, Estonian University of Life Sciences, 51014 Tartu, Estonia; 9 Department of Chemistry and Biotechnology, Tallinn University of Technology, 12618 Tallinn, Estonia

**Keywords:** Vitrification, Ovarian tissue cryopreservation, Follicles, Transcriptomics, In vitro follicle culture, Fertility preservation

## Abstract

**Purpose:**

Ovarian tissue cryopreservation is vital for fertility preservation, yet its effect on ovarian tissue follicle survival and transcriptomic signature requires further investigation. This study delves into the effects of vitrification on tissue morphology, function, and transcriptomic changes, helping to find possibilities for vitrification protocol improvements.

**Methods:**

Ovarian cortex from 19 bovine animals were used to conduct pre- and post-vitrification culture followed by histological assessment, immunohistochemistry, and TUNEL assay. Follicles’ functionality was assessed for viability and growth within the tissue and in isolated cultures. RNA-sequencing of ovarian tissue was used to explore the transcriptomic alterations caused by vitrification.

**Results:**

Follicle density, cell proliferation, and DNA damage in ovarian stroma were unaffected by vitrification. However, vitrified cultured tissue exhibited reduced follicle density of primordial/primary and antral follicles, while freshly cultured tissue manifested reduction of antral follicles. Increased stromal cell proliferation and DNA damage occurred in both groups post-culture. Isolated follicles from vitrified tissue exhibited similar viability to fresh follicles until day 4, after which the survival dropped. RNA-sequencing revealed minor effects of vitrification on transcriptomic signatures, while culture induced significant gene expression changes in both groups. The altered expression of WNT and hormonal regulation pathway genes post-vitrification suggests the molecular targets for vitrification protocol refinement.

**Conclusion:**

Vitrification minimally affects tissue morphology, follicle density, and transcriptomic signature post-thawing. However, culture revealed notable changes in vitrified tissue samples, including reduced follicle density, decreased isolated follicle survival, and alteration in WNT signalling and ovarian hormonal regulation pathways, highlighted them as possible limitations of the current vitrification protocol.

**Supplementary Information:**

The online version contains supplementary material available at 10.1007/s10815-024-03038-4.

## Introduction

Chemo- and radiotherapy, commonly used in cancer treatment, may harm oocytes and follicles, leading to early menopause or primary ovarian insufficiency [1–3], with the extent of damage varying under different conditions [4, 5], emphasizing the need for reliable fertility preservation protocols. Current options like oocyte and IVF embryo freezing have been the most clinically feasible options for preserving women’s fertility [6–8]; however, they pose drawbacks such as potential treatment delays, uncertainty in obtaining sufficient mature oocytes, and inapplicability for prepubertal girls [9].

Ovarian tissue cryopreservation (OTC) was introduced in 2004 [10] to address these challenges, and has resulted in the birth of over 200 babies by 2020 [4]. OTC can be performed using either slow freezing or vitrification. In the slow freezing method, ovarian tissue is exposed to cryoprotectants and gradually frozen down to − 140 °C using a controlled rate freezer. The tissue is then immersed in liquid nitrogen for storage, posing a risk of ice crystal formation and subsequent damage to the ovarian cells, though severe deformation damages have not been reported for thawed tissue samples to date [11, 12].

In contrast, vitrification employs a higher concentration of cryoprotectants than slow freezing and is characterised by a reduced processing time, eliminating the need for expensive equipment. While the primary advantage lies in minimizing the risk of ice crystal formation based on the knowledge from oocyte and embryo vitrification [12], information on the effects of vitrification on ovarian tissue remains limited. Despite its benefits, only four babies have been born using the ovarian tissue vitrification approach [13, 14], and it has yet to find its way into widespread clinical use.

Many research and clinical centres employ diverse ovarian tissue cryopreservation protocols for both slow freezing and vitrification. The lack of universally approved standards poses a challenge in this regard [15–17]. Investigating the effects of vitrification techniques on human ovarian tissue remains challenging due to limited access to such tissue samples. Bovine ovarian tissue has proven to be a valuable animal model in addressing these challenges, as it is readily available from slaughterhouses and shares numerous similarities with human ovaries from folliculogenesis to individual chronological age effects [18–21]. For instance, bovine oocytes and embryos have been used in various IVF studies requiring micromanipulations, while bovine ovaries consistently exhibit analogous reproductive physiology in terms of size, morphology and consistency of ovaries, duration of folliculogenesis, presence of dominant follicle, and follicular size [21, 22].

OTC also encounters challenges, as it may reintroduce malignant cells upon auto-transplantation, particularly in patients with chronic myeloid leukaemia or ovarian cancer [23]. Therefore, the separation of follicles from ovarian tissue and their cultivation in either a two-dimensional format [24] or a three-dimensional in vitro culture system [25], to mature oocytes for IVF, could diminish these risks. Experiments across various species, including rodents [25–27], domestic animals [28–30], non-human primates [31], and humans [32–35], have sought to replicate the ovarian microenvironment; however, only one mouse study has yielded live offspring [27]. Moreover, most of the studies have used fresh ovarian tissue for follicle isolation, while only one study focused on the growth of isolated follicles from cryopreserved tissue [34], without focusing on follicle viability. Nevertheless, it remains unclear whether vitrified and thawed ovarian cortical tissue can yield viable follicles for successful in vitro culture.

In the current study, bovine ovarian cortical tissue served as a model to test the hypothesis that vitrification of ovarian cortical tissue could be an effective method for preserving ovarian tissue with viable follicles. Our study aimed to broaden the understanding of the impact of vitrification by presenting, for the first time, the combination of in-depth morphological and transcriptomic differences that vitrification and in vitro culturing may have on mammalian ovarian tissue, as well as on the viability of isolated follicles derived from both fresh and vitrified ovarian tissue. Moreover, we report, for the first time, vitrification-induced transcriptomic changes that could be used to improve vitrification protocols. Thus, our research provides evidence regarding the robustness of vitrification technology in female fertility preservation and reveals ways to improve the existing vitrification protocols.

## Material and methods

### Collection of bovine ovarian tissue and overall experimental flow

**Fig. 1:**
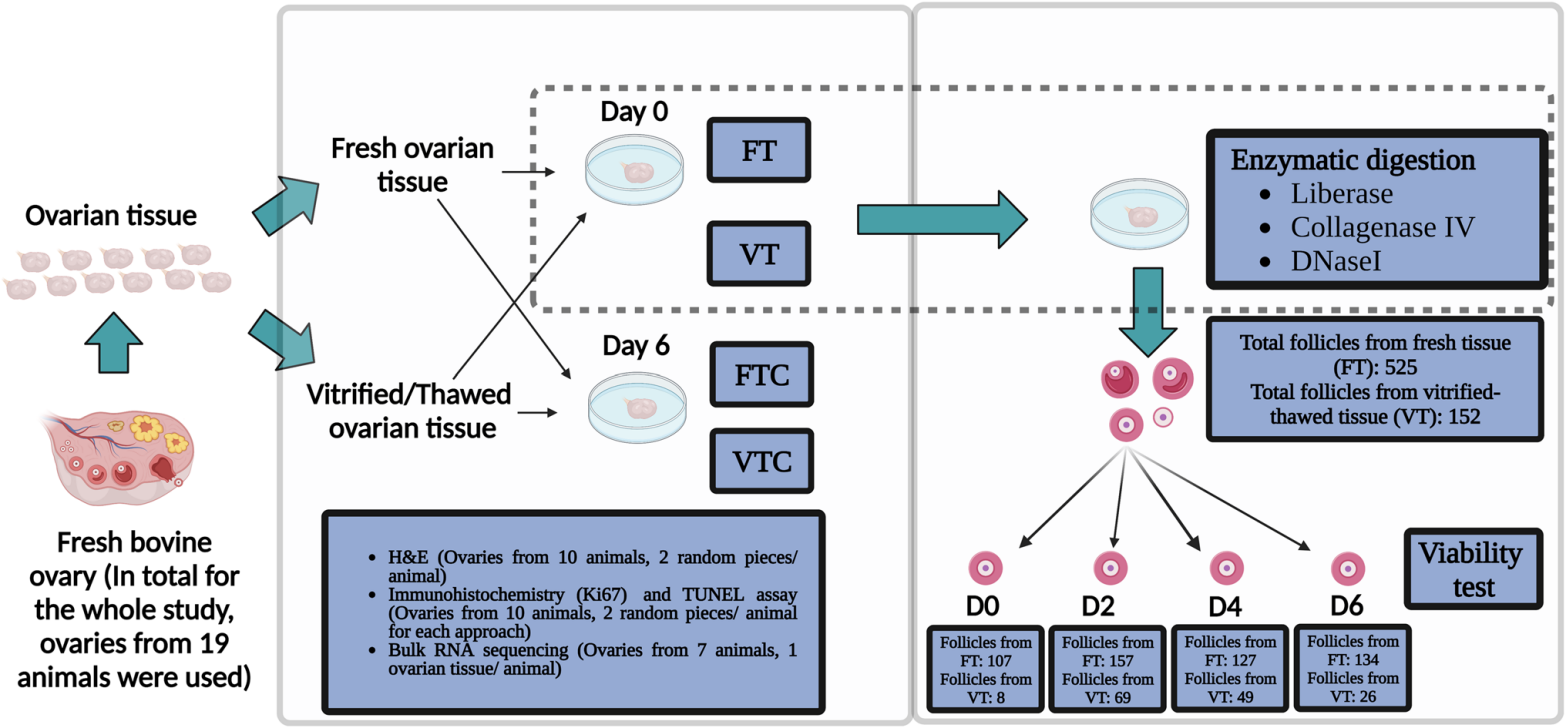
Schematic overview: Bovine ovarian tissue from 19 animals was used for the study. After separating the cortex and medulla, the cortex was used for the study and was divided into four different groups: fresh tissue, FT; fresh tissue cultured for 6 days, FTC; vitrified-thawed tissue, VT; vitrified-thawed tissue cultured for 6 days, VTC. These ovarian tissue pieces were used for hematoxylin and eosin staining (H&E; *n* = 10 animals); immunohistochemical approach with Ki67 antibody (*n* = 10 animals); TUNEL assay (n = 10 animals); and RNA-sequencing (*n* = 7 animals). For FT and VT, tissue pieces from all 19 animals were used for enzymatic isolation of follicles and their follicular culture at various time points (day 0, *n* = 3 animals; day 2, *n* = 3 animals; day 4, *n* = 3 animals; and day 6, *n* = 10 animals) to observe the follicles’ survival using the LIVE/DEAD Test. The figure has been performed using BioRender

Bovine ovaries from 19 animals (2–8 years old) were collected and transported within 1 h at ~ 37 °C in pre-equilibrated L-15 Leibowitz medium (Sigma-Aldrich) from the local slaughterhouse. The ovarian cortex (depth ~ 1–1.5 mm) was separated from the medulla, cut into smaller pieces (~ 2 × 5 × 1–1.5 mm) and used for the following experimental approach outlined in Fig. 1.

In brief, our study involved a comparison between fresh tissue (FT) and the vitrified-thawed tissue (VT) pieces of the ovarian cortex. Additionally, we examined the 6-day fresh tissue with culture (FTC) and vitrified tissue with culture (VTC) in comparison to their uncultured condition. Ovarian tissue pieces from these four different groups were used for follicle counting and evaluation employing hematoxylin and eosin (H&E) stained tissue sections. Cell proliferation was assessed by using Ki67 staining, apoptosis through TUNEL assay, and transcriptomic changes through RNA-sequencing. Finally, we isolated follicles from FT and VT ovarian cortical pieces and evaluated their survival in culture for up to 6 days.

### Cortical tissue collection, vitrification, and thawing

Ovarian cortical pieces were used for the vitrification process and subsequent thawing as described earlier [36] with slight modifications. In summary, the cortical pieces were immersed twice in vitrification solution, containing 40% ethylene glycol (EG, v/v; ThermoFisher Scientific); 30% Ficoll 70 (w/v; Sigma-Aldrich); and 0.5 M sucrose (Sigma-Aldrich), supplemented with 10 mg/ml bovine serum albumin (BSA, Sigma-Aldrich), for 2 and 3 min at room temperature (RT), respectively, transferred into Nunc™ CryoTube™ Vials (ThermoFisher Scientific) and immersed for storage in liquid nitrogen for 2–4 weeks.

For the thawing procedure, the cryovials were removed from the liquid nitrogen, allowed to equilibrate for 30 s at RT, and then incubated in a water bath at 37 °C until melted. The thawed tissue pieces were then placed into decreasing concentrations of thawing solution (1 M and 0.5 M Sucrose; Sigma-Aldrich) diluted in basic solution (Phosphate-buffered saline, PBS-/-; ThermoFisher Scientific) supplemented with Glucose (Sigma-Aldrich) and Pyruvate (Sigma-Aldrich) for 2 and 3 min, respectively, and washed in a basic solution containing 10 mg/ml of BSA (Sigma-Aldrich) for 5 min.

#### Tissue culture and fixation

Two random replicates from fresh and vitrified tissue pieces were fixed overnight in Bouin’s solution (VWR Chemicals, Belgium), 4% paraformaldehyde (PFA; ThermoFisher Scientific) and RNAlater (ThermoFisher Scientific) at − 80 °C. Additionally, two random replicates from fresh and vitrified ovarian cortical pieces were cultured in 96-well plates containing 150 μl of αMEM culture media supplemented with 0.1 mg/ml penicillin–streptomycin (ThermoFisher Scientific); 1 × insulin–transferrin–selenium solution (ITS; ThermoFisher Scientific); 50 mg/ml l-ascorbic acid; 10 mg/ml of BSA (Sigma-Aldrich), and 3 mM glutamine (ThermoFisher Scientific), for 6 days at 37 °C in humidified air incubator with 5% CO_2_. Half of the culture media was changed every 2 days, and at the end of the culture period, the tissue pieces were fixed as described above.

### Histology analysis

Fixed ovarian tissue pieces from 10 animals, two random replicates from each, were dehydrated in gradually increasing ethanol concentration, followed by double xylene incubation for 1.5 h and embedded into paraffin. Sections of 4-μm thickness were obtained using a rotary microtome (MICROM, HM 355S; ThermoFisher Scientific).

H&E staining was conducted according to a standard protocol [37], and the slides were subsequently scanned at 20 × magnification using a slide scanner (Leica SCN400). The images were visualised by Aperio ImageScope software (Leica Biosystems).

The developmental phases of the follicles were classified based on the morphology of their granulosa cells (GCs) and categorised as follows: (1) monolayered follicles: characterised by a single layer of either flattened or cuboidal GCs surrounding the oocyte; (2) secondary follicles, where the oocyte is surrounded by multiple layers of GCs; and (3) antral follicles characterised by the presence of an antrum surrounded by a multilayer of GCs [20]. Additionally, follicles with pyknotic oocytes or GCs were classified as atretic follicles [20]. Two random sections were selected from each replicate, and two researchers blindly counted the follicles. To prevent double counting, only follicles with a visible nucleus were included in the count. Follicle density was calculated by dividing the number of follicles by the counted tissue area.

#### Immunohistochemistry analysis for Ki67

Unless otherwise indicated, all washes were performed 3 times in PBS (Sigma-Aldrich), each lasting for 5 min, and all the dilutions were carried out in 1% BSA/PBS.

To assess the proliferating cells within the ovarian tissue, we used an immunohistochemical approach with a monoclonal mouse/rat anti-human Ki67 (SolA15) antibody (Invitrogen, ThermoFisher Scientific). Two random sections obtained from two random ovarian tissue pieces for each one of the 10 animals were used. These sections were deparaffinised and rehydrated by gradually decreased ethanol (EtOH) concentration, followed by heat-mediated antigen retrieval at ~ 98 °C with 10 mM sodium-citrate buffer pH = 6.0 for up to 20 min. For permeabilization, sections were incubated for 20 min in 0.1% Triton- × 100, blocked for 1 h in 5% goat serum and followed by overnight incubation with primary antibody at a 1:200 dilution at 4 °C, while the negative control was incubated in 1% BSA/PBS. Following the primary antibody incubation, the sections were washed with PBS and incubated with goat anti-mouse Alexa Fluor 488 secondary antibody (1:1000, ThermoFisher Scientific) for 1 h, at RT. DAPI (4’,6-diamidino-2-phenylindole dihydrochloride; Sigma Aldrich) was used as a nuclear counterstain, followed by washes. Slides were mounted using Fluoromount™ Aqueous Mounting Medium (Sigma-Aldrich) and were stored at 4 °C for further analysis.

The slides were visualised at 10 × magnification, and the Ki67 positive (Ki67 +) cells were blindly counted. The density of the Ki67 + cells was calculated by dividing the Ki67 + cells by the ovarian tissue area, and the data were reported in arbitrary normalised units.

### TUNEL assay

DeadEnd™ Fluorometric TUNEL System (Promega) was used to identify fluorescein-12-dUTP–labelled fragmented DNA following the manufacturers’ guidelines.

In brief, two random 4% PFA-fixed ovarian tissue pieces from 10 animals and two random sections from each replicate were deparaffinised and rehydrated, followed by incubation in equilibration buffer at RT for 15 min. Sections were subsequently incubated with a solution containing equilibration buffer, biotinylated nucleotide mix, and recombinant terminal deoxynucleotidyl transferase (rTdT) enzyme at 37 °C for 60 min. The negative control was incubated in a solution containing equilibration buffer, biotinylated nucleotide mix and Milli-Q water. The reaction was terminated by 2 × saline sodium citrate (SSC) at RT for 15 min. DAPI was used as a nuclear counterstain, followed by PBS wash. Slides were mounted using Fluoromount™ Aqueous Mounting Medium and stored at 4 °C for further analysis.

The slides were visualised at 10 × magnification, and the cells positively stained for TUNEL per tissue area were semi-automatically counted using the Fiji (ImageJ) TUNEL Cell Counter tool, following the guidelines described previously [38]. Cells positively stained for TUNEL were counted, and their density was calculated by dividing them by the ovarian tissue area. The data were reported in arbitrary normalised units.

### Isolation and culturing of follicles from ovarian cortical tissue

For the isolation of single follicles, 20 pieces of ovarian cortical tissue from a total of 19 animals were used, following the previously described method for follicle isolation [33], with slight modifications. Briefly, FT and VT pieces were manually chopped into 0.1 mm^3^ pieces in 37 °C pre-incubated handling media, McCoy 5A with 25 mM HEPES (ThermoFisher Scientific) supplemented with 0.5 mg/ml BSA (Sigma-Aldrich), 2 mM GlutaMAX (ThermoFisher Scientific), 100 U/ml penicillin/streptomycin (ThermoFisher Scientific), and 1 × insulin-transferrin-selenium (ITS; ThermoFisher Scientific).

Next, the homogenised tissue pieces were placed in 7.5 ml of digestion media, including handling media supplemented with 50 mg/ml of Neutral Red (NR; Sigma-Aldrich), 0.04 mg/ml Liberase™ (Sigma-Aldrich), 0.2 mg/ml collagenase IV (ThermoFisher Scientific), and 0.2 mg/ml deoxyribonuclease I (DNase I; Roche) and incubated with gentle agitation at 37 °C for 45–60 min. The enzymatic digestion was terminated by adding an equal amount of pre-equilibrated handling media supplemented with 10% fetal bovine serum (FBS; ThermoFisher Scientific) and 100 U/ml penicillin/streptomycin (ThermoFisher Scientific).

The NR-stained live follicles were collected using an EZ-range type stripper pipette (Cooper Surgical) and washed in handling media before encapsulation in 0.5% alginate bead (Sigma-Aldrich). Subsequently, 4–7 NR-stained follicles were transferred into the alginate solution and dropped into the cross-linking solution (140 mM NaCl and 50 mM CaCl_2_ dissolved in Milli-Q H_2_O) to form a bead. After 2 min, the alginate beads containing the encapsulated live follicles were washed in pre-equilibrated culture media, αMEM culture media (Sigma-Aldrich) supplemented with 0.1 mg/ml penicillin–streptomycin (ThermoFisher Scientific), 1 × insulin–transferrin–selenium (ITS; ThermoFisher Scientific), 50 mg/ml l-ascorbic acid (Sigma-Aldrich), 100 ng/ml rAct-A (Bio-Techne), 50 ng/ml rhFSH (ProSpec), 3 mg/ml of BSA (Sigma-Aldrich) for 15 min and transferred to a 96-well plate containing 150 μl of culture media at 37 °C in 5% CO_2_ incubator for up to 6 days of culture. Half of the culture media was changed every second day with a pre-equilibrated 37 °C culture media. All the solutions were prepared fresh every second week and filtered using 0.22 μm GVS syringe filters (Novatech International).

#### Follicle viability assessment using LIVE/DEAD staining

The viability of the follicles from FT and VT was assessed immediately after isolation and on days 2, 4, and 6 of the culture using double fluorescent labelling with calcein AM and ethidium homodimer (EthD-1) dyes (LIVE/DEAD™ Viability/Cytotoxicity Kit for Mammalian Cells; ThermoFisher Scientific) as described previously [39].

During the viability assessment, the alginate encapsulated follicles were incubated in 1.6 mM calcein AM and 0.5 mM EthD-1 for 20–45 min at 37 °C in the dark. Following the incubation period, follicle viability was evaluated under a fluorescent microscope (Nikon, Eclipse TS100) by assessing green (calcein = viable) and red (EthD-1 = dead) labelling which was used to classify the follicles as follows. Follicles with green oocytes and at least 60% viable GCs were classified as alive, while follicles with red oocytes and over 60% dead GCs were classified as atretic.

### RNA sequencing

#### RNA extraction and DNase treatment

Bovine ovarian pieces from 7 animals for each group were used for RNA extraction using the RNeasy mini kit (Qiagen). The tissue was initially disrupted using scalpels and then placed into 600 μl of buffer RLT mixed with β-mercaptoethanol and homogenised using TissueRuptor (Qiagen). The homogenate was centrifuged for 3 min at full speed to separate the supernatant and the pellet. The supernatant was mixed with an equal volume of 70% EtOH and then transferred into the RNeasy spin column and centrifuged for 15 s at 8000 g. The spin column was washed with buffer RW1 and buffer RPE with each wash followed by 15 s centrifugations at 8000 g. Another wash with buffer RPE was performed but with more prolonged centrifugation (2 min at 8000 g) to ensure complete drying of the spin column and prevent any EtOH carryover. To minimise potential residues outside the column, 1 min centrifugation at full speed was performed. The RNA was eluted by adding 30 μl of RNase-free water followed by 1 min centrifugation at 8000 g.

To eliminate contaminating genomic DNA from RNA samples, DNase treatment was performed using Ambion® DNA-free™ DNase Treatment and Removal Reagents (ThermoFisher Scientific). The RNA was mixed with 0.1 volume of 10 × DNase I buffer and 1 μl of rDNase I and incubated for 30 min at 37 °C. After incubation, the treatment was deactivated using 0.1 volume of DNase Inactivation Reagent with occasional mixing during the 2 min incubation period at RT. Subsequently, centrifugation was performed at 10,000 g for 1.5 min to separate the RNA (supernatant) from the reagents (pellet).

The concentration and integrity of the purified RNA were assessed using the NanoDrop™ 2000/2000c spectrophotometer (ThermoFisher Scientific) and 2100 Bioanalyzer with the RNA 6000 Pico Kit (Agilent Technologies, Santa Clara, CA, USA), respectively. Samples with RNA integrity number (RIN) > 7 (RIN range 7–9.2) were used for the preparation of the libraries.

#### Library preparation

Sequencing libraries were prepared from the purified RNA using the NEXTFLEX Rapid Directional RNA-seq Kit 2.0 (Perkin Elmer). To enable multiplexing, samples were indexed, and the libraries’ quality and size range were assessed using a 2100 Bioanalyzer with the high sensitivity DNA kit (both Agilent Technologies). Then the libraries were diluted to a final concentration of 2 nM and sequenced on an Illumina HiSeq 4000 platform with single-end reads of 75 bp, ensuring a minimum of 13 million reads per sample.

#### Quality control, alignment, and quantification of the sequencing reads

Raw reads in FASTQ format were processed with the FASTQC [40] program (version 0.11.8) to assess the overall read quality. The FASTP program [41] was used for read trimming and removal of adaptor sequences. The resulting trimmed reads were aligned to the *Bos Taurus* genome assembly (ARS-UCD1.2) obtained from the Ensembl database [42]. This alignment with the reference genome was performed using the STAR program [43] with default parameters. Gene counts were generated using the featureCounts program [44] with default parameters.

#### Differential gene expression analysis

The analysis of differentially expressed genes (DEGs) was performed on the count data using the DESeq2 R package [45]. Genes with minimum of ten counts for all the samples in at least one of the experimental groups were retained in the analysis. Pairwise differential gene expression analysis was performed between: (a) VT vs. FT, (b) FTC vs. FT, (c) VTC vs. FTC, and (d) VTC vs. VT. The resulting *p* values were adjusted using Benjamini and Hochberg’s (BH) approach [46] for controlling the false discovery rate (FDR). Genes with an absolute fold change |FC|> 2 and FDR-adjusted *p* value (*q* value) < 0.05 were considered as differentially expressed.

Principal component analysis (PCA) was performed using the plotPCA function from DESeq2 package [45]. Prior to the PCA, we performed variance stabilising transformation (VST) on the raw count data to reduce any potential biases in the clustering analysis.

#### Gene set enrichment analysis

For pathway analysis we utilised the g:Profiler web tool [47] adapting the analysis specifically for *Bos Taurus*. The g:GOSt function was utilised for gene-set enrichment analysis, and significance was determined using the tailored significance threshold g:SCS adapted by g:Profiler. Pathways with an adjusted *p* value below 0.05 were considered statistically significant. To visualise the KEGG pathways we utilised the Bioconductor package pathway version 1.43.0 providing a comprehensive representation of the enriched pathways.

### Statistical analysis

Data from the experiments were analysed using GraphPad Prism 9 (GraphPad Software Inc.). A two-way ANOVA with multiple comparisons was applied to evaluate the statistical significance between FT and FTC, VT, and VTC, and a *p* value < 0.05 was considered statistically significant. All the data were reported in arbitrary normalised units. Additionally, a one-way ANOVA with multiple comparisons was applied for the isolated follicles to assess the statistical significance among the follicles that survived during in vitro culture on days 2, 4, and 6; in comparison to those that survived on day 0.

## Results

### Study setup

The study setup is outlined in Fig. 1. Bovine ovaries were used to investigate the impact of vitrification on tissue and follicle morphology, viability upon culture and gene expression. A total of 19 ovarian samples from 19 animals were collected. Samples from 10 animals were divided into four groups for the tissue culture experiment: fresh tissue and fresh tissue with culture (FT and FTC) as well as vitrified tissue and vitrified tissue with culture (VT and VTC). Samples were harvested for immunohistochemistry and RNA-sequencing. In parallel, ovaries from 19 animals were used for isolated follicle culture experiments, where follicles extracted from FT and VT groups were followed in culture for 6 days and assessed for viability on days 0, 2, 4, and 6 (Table 1).

**Table 1:** Overall animal and sample information for the study. Bovine ovaries yielded sufficient material for multiple experiments, and as a result, the same animal samples were utilised in multiple experiments. The table displays the number of animals that provided ovarian tissue for all four study groups in hematoxylin and eosin staining (H&E), immunohistochemistry, TUNEL assay, and RNA sequencing

Conditions	Fresh tissue	Vitrified-thawed tissue
Total number of animals providing ovaries (*n*)	19	19
Number of animals used for ovarian tissue experiments	10	10
Total number of animals used to collect ovaries for tissue culture, H&E, immunohistochemistry and TUNEL assay (*n*)	10 (2 tissue pieces from each animal)	10 (2 tissue pieces from each animal)
Total number of animals used to collect ovaries for RNA extraction, library preparation and RNA sequencing (*n*)	7 (1 tissue piece from each animal)	7 (1 tissue piece from each animal)
Number of animals used for the isolation of follicles	19	19
Total number of follicles (*n*) isolated from ovaries collected from 19 animals	525	152
Number of isolated follicles (*n*) for day 0 viability test from three animals	107	8
Number of isolated follicles (*n*) for day 2 viability test from three animals	157	69
Number of isolated follicles (*n*) for day 4 viability test from three animals	127	49
Number of isolated follicles (*n*) for day 6 viability test from ten animals	134	26

### Histological evaluation of ovarian tissue cortical pieces

**Fig. 2:**
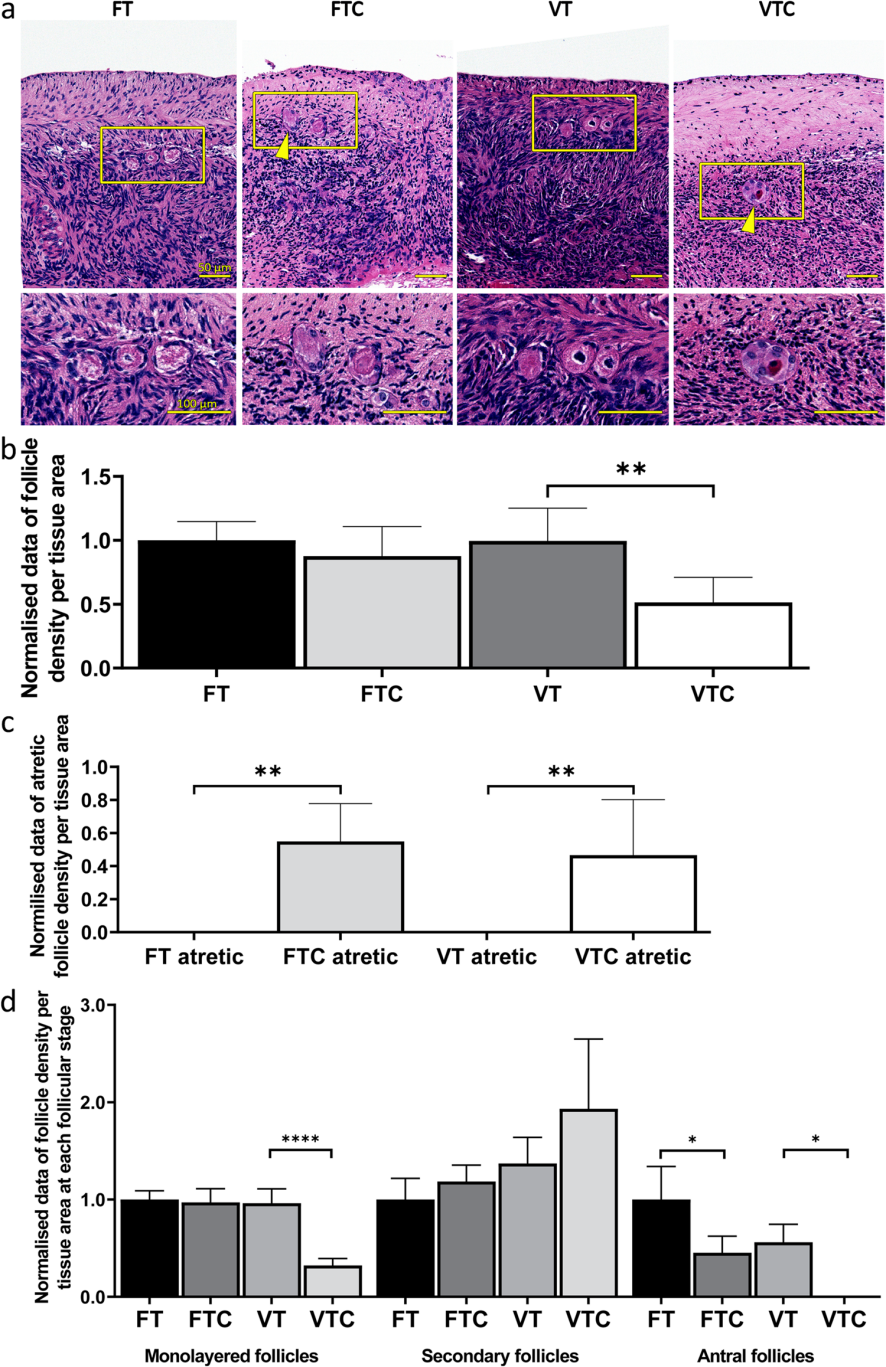
Histological evaluation of ovarian tissue: **a** Representative histological H&E-stained images from the experimental groups fresh tissue (FT); fresh tissue with culture (FTC); vitrified-thawed tissue (VT); and vitrified-thawed tissue with culture (VTC). Yellow arrowheads point to atretic follicles. Yellow rectangles indicate a higher magnification of the tissue presented in the lower row of images. Scale bars 50/100 μm. **b** Bar graph showing the normalised density of viable follicles (sum of monolayered, secondary and antral follicles) in the different groups. Four random sections per animal were used to calculate the follicle density, and only follicles with visible nucleus were included in the calculation. **c** Normalised density of atretic follicles, and **d** normalised density of follicles by developmental stage. Data are shown as average ± SEM (10 animals per group). Two-way ANOVA. *P ≤ 0.05, **P ≤ 0.01, ****P ≤ 0.0001

A histological assessment was performed to examine differences in follicle density and morphological changes among the four groups (Fig. 2a). Follicles were categorised into four groups: monolayered, secondary, antral, and atretic follicles. The impact of vitrification and culturing was histologically assessed by evaluating the morphology of the follicles and the rest of the tissue [48]. As illustrated in Fig. 2a, the FT exhibited ovarian tissue morphology characterised by properly organised granulosa and stromal cells with the absence of atretic follicles. In contrast, FTC displayed a similar overall morphology with normal stromal cells and follicles; however, there was a significant increase (*p* value = 0.0017) in the number of follicles with pyknotic or disorganised granulosa cells (atretic follicles) (Fig. 2a, FTC yellow arrowheads; quantified in Fig. 2c), when compared with FT. The assessment of VT revealed normal follicles and stromal cells, and the absence of atretic follicles (Fig. 2a, VT; quantified in Fig. 2c). Finally, the density of healthy follicles, excluding atretic follicles, was significantly reduced and that of atretic follicles increased compared to VT control (Fig. 2a, VTC yellow arrowheads; quantified in Fig. 2b, p value = 0.0051; Fig. 2c, p value = 0.0016).

The follicles were further sub-categorised per growth stage to monolayered (includes primordial and primary follicles), secondary and antral follicles for statistical analysis. We found no differences in the density of monolayered follicles between FT, FTC, and VT. However, there was a significant reduction in the density of monolayered follicles in the VTC group (*p* value < 0.0001; Fig. 2d). We did not find any significant difference in the density of secondary follicles among all four groups (Fig. 2d). For antral follicles, a significant decrease was observed in FTC (*p* value = 0.0122; Fig. 2d) and in VTC (*p* value = 0.0190; Fig. 2d) groups compared to FT and VT, respectively.

### Culturing the ovarian tissue increased the proliferation and the number of DNA double-stranded breaks in ovarian stromal cells

**Fig. 3:**
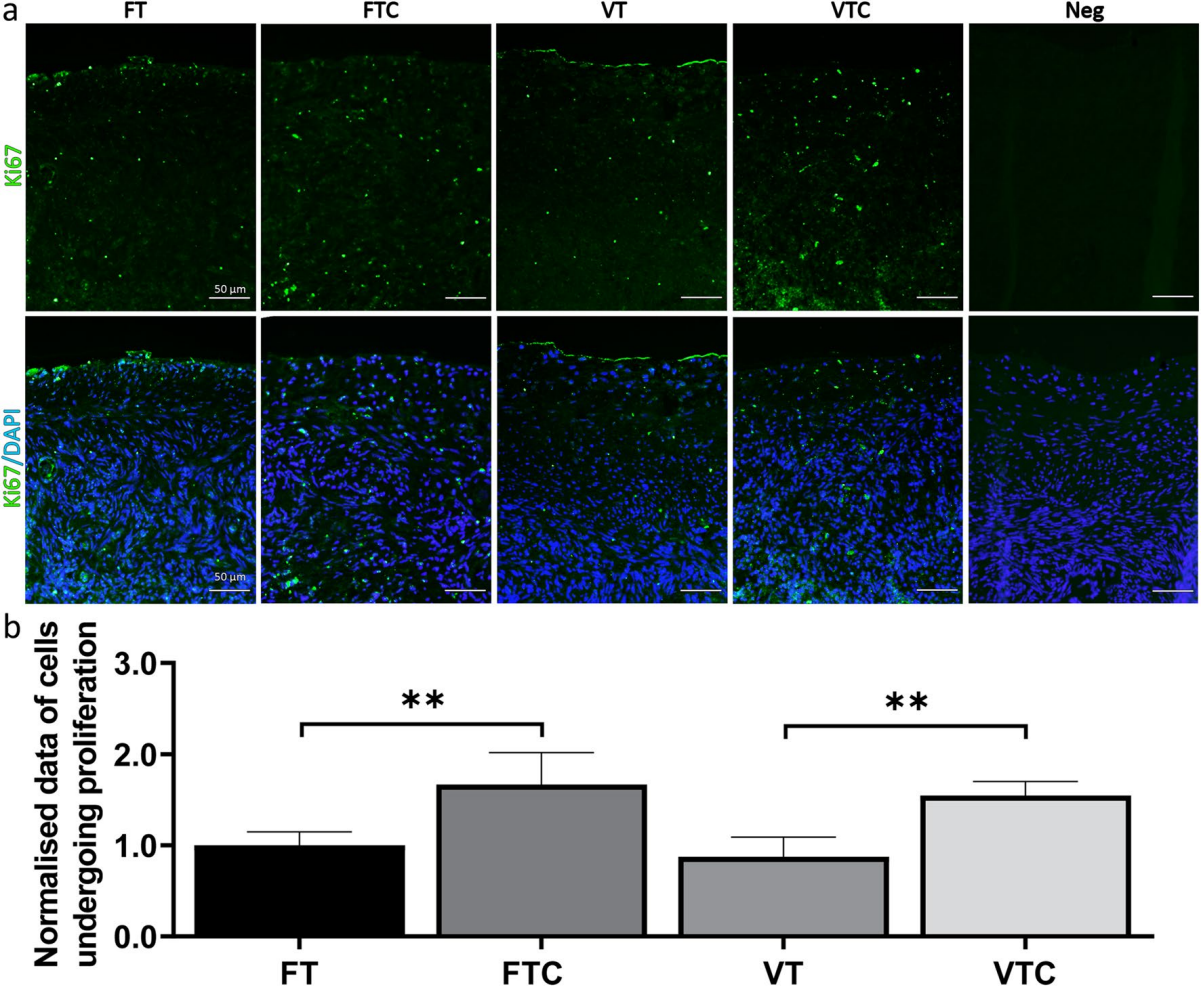
Cell proliferation before and after culture: **a** Representative images showing Ki67 positive cells in green and nuclear DAPI-stain in blue across the different experimental groups, **b** Quantification of the Ki67 positive cells presented as a bar graph. For this analysis, four random sections per animal were used to calculate the number of Ki67 positive cells, and the count was normalised to the analysed tissue area. Data is shown as average ± SEM (10 animals per group). Scale bars 50 μm. Two-way ANOVA. **P ≤ 0.01

To investigate the impact of vitrification and culture on cell proliferation, we conducted immunohistochemical staining using monoclonal mouse/rat anti-human Ki67 antibody. The analysis showed no statistical differences in the density of the proliferating cells between FT and VT (*p* value = 0.8605; Fig. 3a, b). Conversely, culture increased the number of proliferative cells in both groups to a similar extent (*p* value = 0.0030 FTC, and *p* value = 0.0027 VTC compared to FT and VT, respectively) (Fig. 3a, b).

**Fig. 4:**
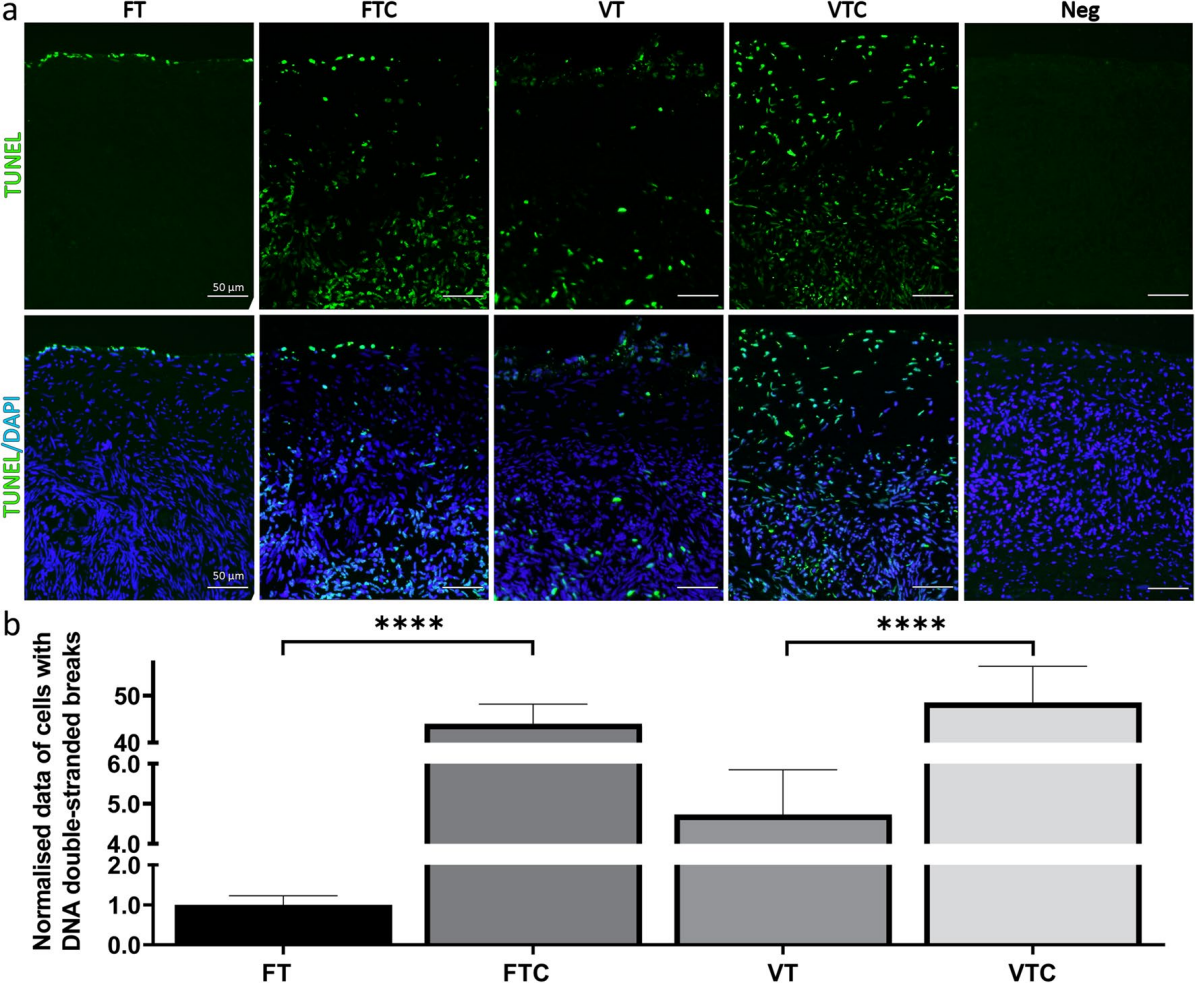
Cell death before and after culture: **a** Representative images showing TUNEL-positive (TUNEL +) cells (green stained) and nuclear DAPI (blue stained) in the different experimental groups. **b** Quantification analysis of TUNEL + cells is presented as a bar graph. For TUNEL + cell counting, four random sections per animal were used to calculate the number of TUNEL positive cells, and the count was normalized to the analysed tissue area. Data is shown as average ± SEM (*n* = 10 animals per group). Scale bars 50 μm. Two-way ANOVA. ****P < 0.0001

To assess the impact of vitrification and culture on DNA damage, we conducted TUNEL assay. Visually, vitrification as such seemed to increase the number of TUNEL positive cells in the tissue; however, this did not reach statistical significance upon quantification (*p* value = 0.6837 for VT to FT) (Fig. 4a, b). In contrast, both culture groups showed a significant increase in TUNEL-positive cells compared to the non-cultured controls (*p* value < 0.0001 for both; Fig. 4a, b).

### The survival rate of isolated follicles during the 6-day culture period

**Fig. 5:**
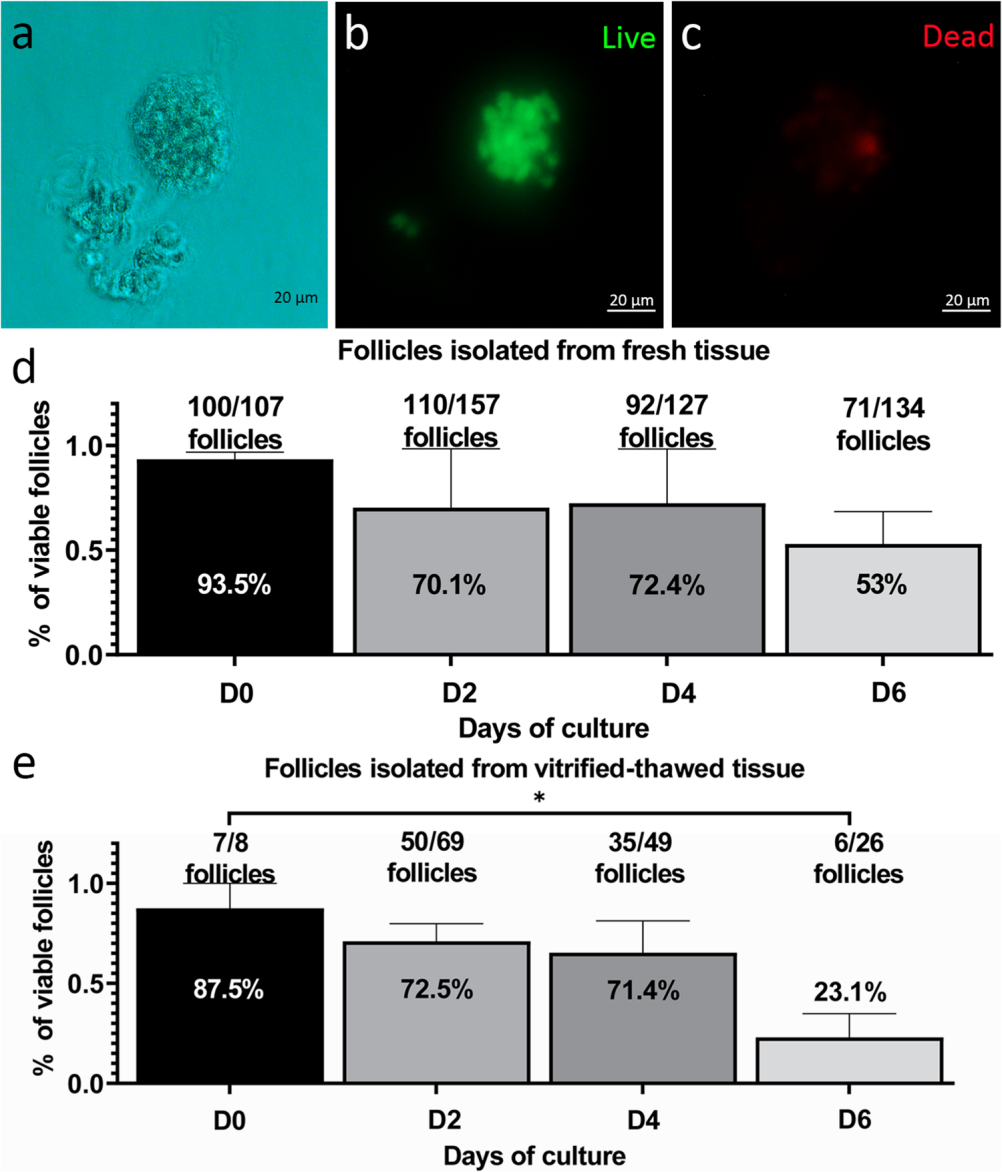
Viability of isolated follicles during culture: **a** Representative bright-field image of a follicle after isolation. **b** Representative image of a live follicle characterized by viable granulosa cells (green). **c** Representative image of a few dead cells (red) in previous follicle. **d** Viability of follicles isolated from fresh tissue (FT) during 6-day culture. Altogether 525 follicles were isolated from fresh tissue and subjected to culture, and subgroups of these follicles were used in viability assessment at the start and on days 2, 4, and 6. **e** Viability assessment of follicles isolated from vitrified tissue (VT) during 6-day culture. Altogether 152 follicles isolated from vitrified tissue were cultured, and subgroups were used for viability assessment at start and on days 2, 4, and 6 of culture. Results of the viability of the different follicles counted are presented as average viability ± SEM (*n* shown above the bars). One-way ANOVA. *P ≤ 0.05

The morphological evaluation suggested no major impacts of vitrification on tissue upon thawing; yet follicular density was significantly reduced during tissue culture in the vitrified group. This finding suggests that possible cryopreservation damage on follicles may become visible only in functional studies like during culture. To test this, we extracted follicles from FT and VT groups and assessed their growth and health during in vitro culture (Fig. 5a). The viability assessment was performed using double fluorescent labelling with calcein AM and EthD-1, where calcein is retained within live cells, generating uniform green fluorescence, and EthD-1 can only cross damaged cell membrane, colouring dead cells by red fluorescence dye. Follicles were characterised either as alive (oocyte and ≥ 60% of GCs green) or dead (oocyte and ≥ 60% of GCs red) (Fig. 5b, c). In total, we successfully isolated 525 cortical follicles from fresh tissue and 152 follicles from their vitrified-thawed counterparts from 19 ovaries. Follicles were assessed every second day of culture.

On day 0, we used 107 follicles from the FT tissue and 8 from the VT for viability testing. There were no differences between the groups, with 100 follicles (93.5%; Fig. 5d) and 7 follicles (87.5%; Fig. 5e) classified as alive, respectively, for FT and VT groups.

The viability of the follicles isolated from FT did not statistically decrease during the culture (day 2, *p* = 0.85; day 4, *p* = 0.8808, and day 6, *p* = 0.3913) (Fig. 5d). Follicles isolated from VT showed similarly high viability up to day 4 of culture (day 2, *p* = 0.8487 and day 4, *p* = 0.7133), but there was a significant reduction thereafter, with only 6 follicles out of 26 remaining alive on day 6 (23.1%; *p* = 0.0199; Fig. 5e).

In conclusion, follicles isolated from vitrified-thawed tissue stay alive and can be successfully cultured until day 4 but have lower viability in vitro since day 6 compared to follicles isolated from fresh tissue.

### Minimal impact of vitrification/thawing on ovarian tissue transcriptome

**Fig. 6:**
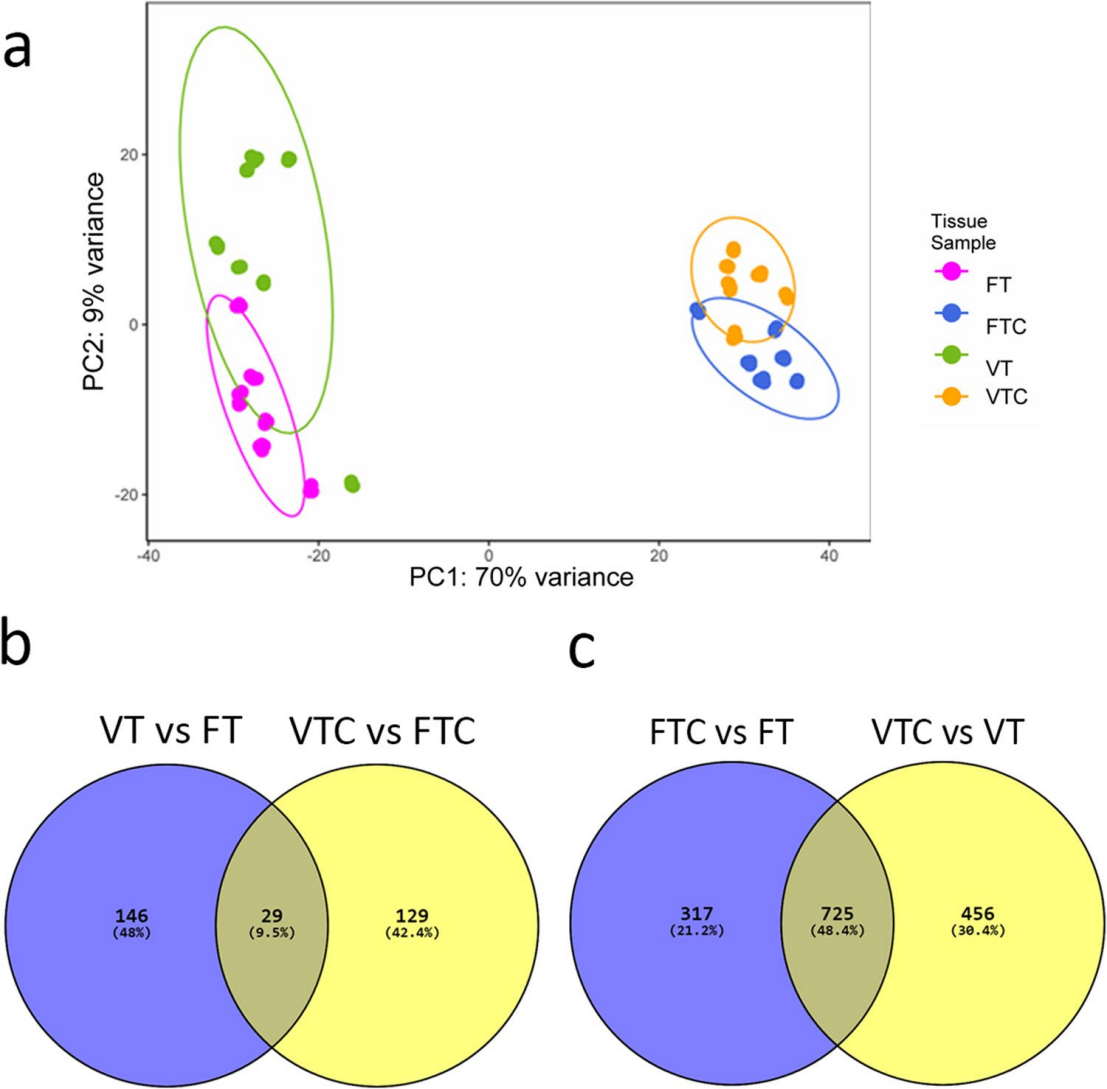
Overview of transcriptomic changes during culture: **a** Principal component analysis (PCA) of bulk RNA expression in ovarian cortical tissue of fresh tissue (FT; purple), fresh tissue with culture (FTC; blue), vitrified-thawed tissue (VT; green), and vitrified-thawed tissue with culture (VTC; orange). Circles in the plot serve to denote confidence regions for each group, offering a visual indication of variance and covariance within conditions. Larger circles signify greater dispersion, whereas smaller ones indicate tighter clustering. **b** Venn diagram representing the overlap of differentially expressed genes (DEGs) between the comparison of fresh tissue and vitrified tissue, i.e., VT vs. FT and VTC vs. FTC. **c** Venn diagram representing the overlap of DEGs between the comparisons of tissues with culture and those without culture, i.e., FTC vs. FT and VTC vs. VT

To gain insights into the impact of vitrification on post-thaw ovarian tissue, transcriptomic analysis was carried out. Altogether one cortical piece per animal from FT, VT, FTC, and VTC covering 7 animals was subjected to bulk RNA-sequencing. The principal comportment analysis (PCA) had a strong PC1 that explained 70% of the variance, dividing the samples into uncultured (FT and VT) and cultured (FTC and VTC) tissue specimens (Fig. 6a). Although, PC2 further separated the samples to fresh and vitrified, this clustering was less evident. In summary, most of the variation in the gene expression data was caused by the culture of fresh and vitrified-thawed tissue samples.

Next, differentially expressed genes (DEGs) were determined as those that differed by |FC|> 2 at FDR < 0.05. Fresh and vitrified ovarian tissue samples (VT vs. FT) differed by 175 DEGs, with vitrification upregulating 128 DEGs (Supplementary Table S1). Vitrified cultured and freshly cultured, i.e., VTC vs. FTC samples, differed by 158 DEGs, where 13 genes were upregulated by vitrification (Supplementary Table S1). The overlap between these two comparisons revealed 29 DEGs, such as *CCNA2*, *GSTA2*, *INSL3*, *IRS4*, *MMP3*, *OVOL1*, and *RELN*, that could represent vitrification related genes that persist in culture (Fig. 6b).

We then focused on culture-related changes and identified 1042 DEGs that changed in freshly collected tissue upon culture (FTC vs. FT) (Supplementary Table S1), and 1181 DEGs that changed in vitrified tissue in culture (VTC vs. VT). In both comparisons, equal number of genes was up- and downregulated (Supplementary Table S1). There was a marked overlap in culture-induced changes in gene expression between these two comparisons (*n* = 725 DEGs); however, there were also hundreds of group-specific DEGs (*n* = 317 DEGs in fresh and *n* = 456 in vitrified tissue) (Fig. 6c).

### Pathway enrichment analysis

To assign functions to the identified groups of DEGs, we conducted a gene set enrichment analysis (GSEA) based on the KEGG annotation for *Bos Taurus* using the g:Profiler web tool and FDR < 0.05 as a cutoff. In the case of the changes related to vitrification (VT vs. FT and VTC vs. FTC), we were unable to find significant pathway enrichments, likely due to the relatively small number of DEGs (*n* < 200).

**Fig. 7:**
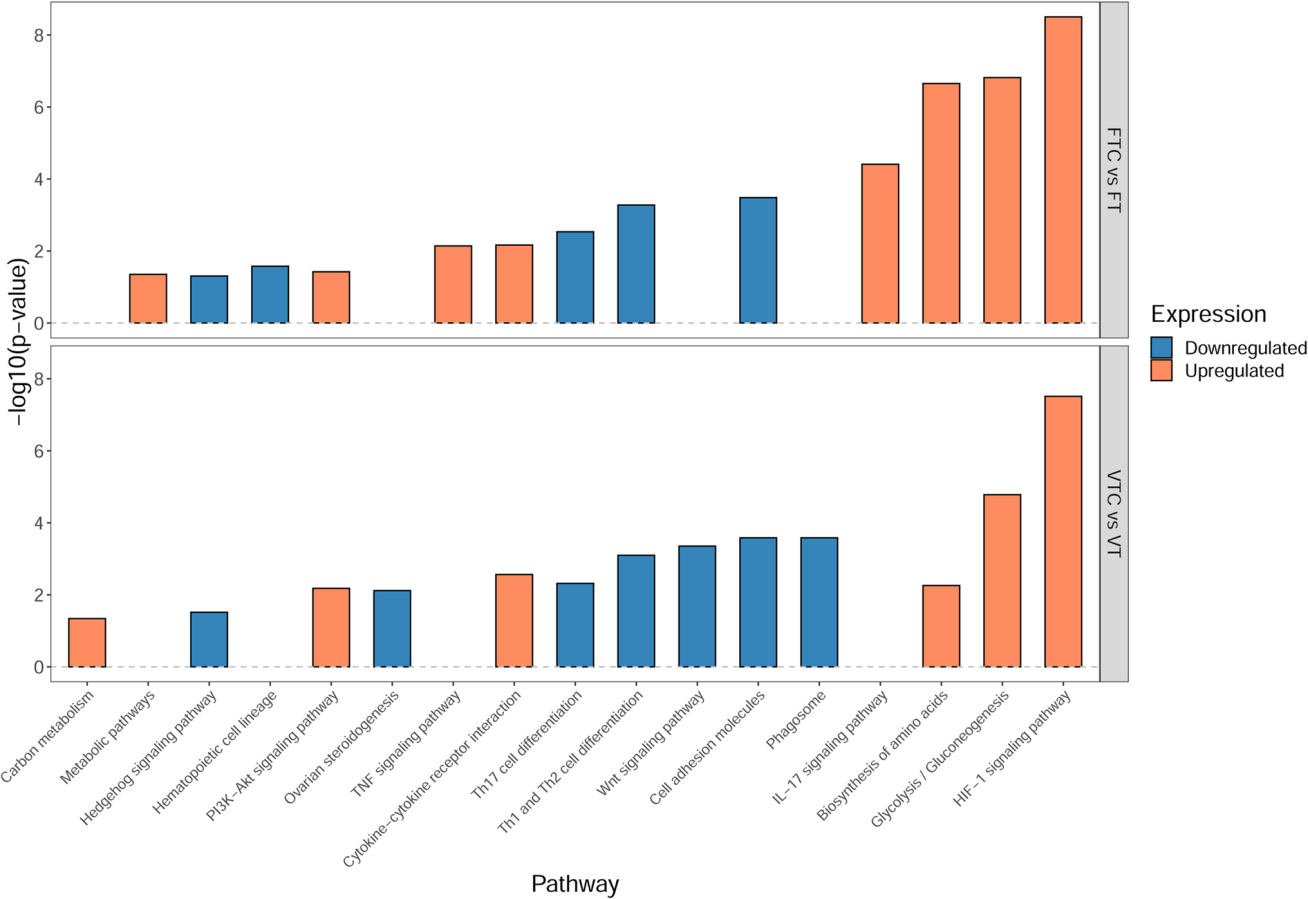
Enriched signalling pathways during culture: Enriched signalling pathways were identified based on gene expression changes during culture for fresh and vitrified tissue groups using KEGG pathways as gene sets. The top enriched pathways were then plotted to compare the groups. Orange and blue bars represent enriched KEGG pathways related to up- and downregulated genes, respectively. The y-axis represents the p-adjusted values using the Benjamini–Hochberg procedure. The x-axis represents the corresponding KEGG pathway. The upper and lower panels are for FTC vs. FT and VTC vs. VT comparisons, respectively

On the contrary, culture-induced changes were enriched for multiple KEGG pathways. In fresh tissue (FTC vs. FT), 24 pathways were associated with upregulated DEGs, and 34 with downregulated DEGs (Supplementary Table S2). The top significant pathways identified encompassed the HIF-1 signalling pathway; glycolysis/glyconeogenesis; and biosynthesis of amino acids (Fig. 7). In the vitrified group, 18 pathways were associated with the DEGs upregulated during culture, and 38 pathways to the downregulated DEGs (Supplementary Table S2). The most significant pathways included the HIF-1 signalling pathway and glycolysis/glyconeogenesis (Fig. 7). We then compared the changes in the two groups to identify pathways that were specifically altered in vitrified tissue in culture. We found that although the most significant pathways were shared between the two cultured groups, there were some changes that only took place in the vitrified tissue, namely Wnt signalling; ovarian steroidogenesis, carbon metabolism, and phagosome pathways (Fig. 7).

**Fig. 8:**
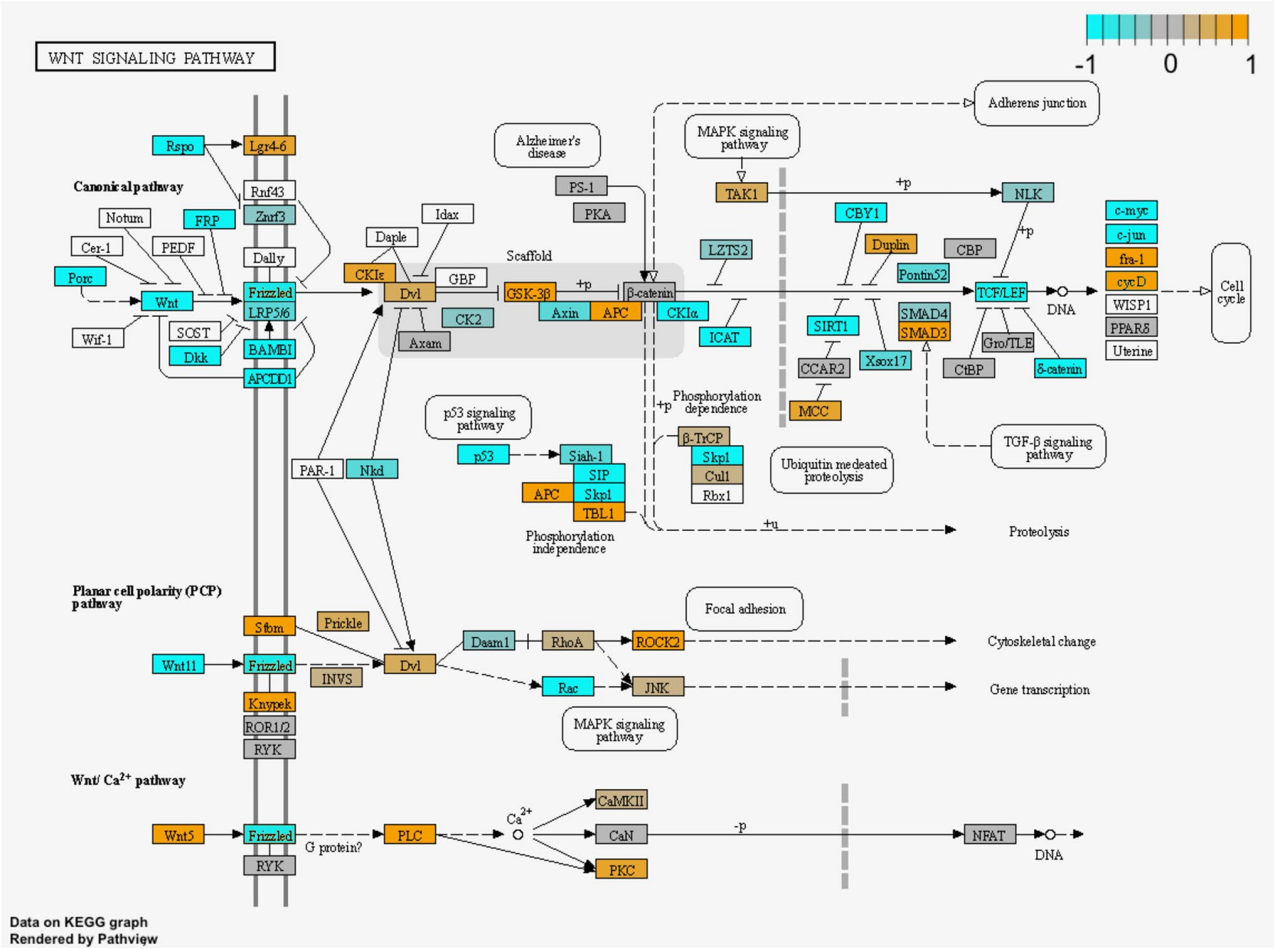
Alteration in WNT signalling during culture of vitrified tissue: Relative gene expression in the KEGG pathway illustrated in orange colour (upregulated gene expression during culture) and blue (downregulated gene expression during culture) for VTC vs. VT comparison. Undetected genes are shown in white. The grey colour indicates no change in expression

**Fig. 9:**
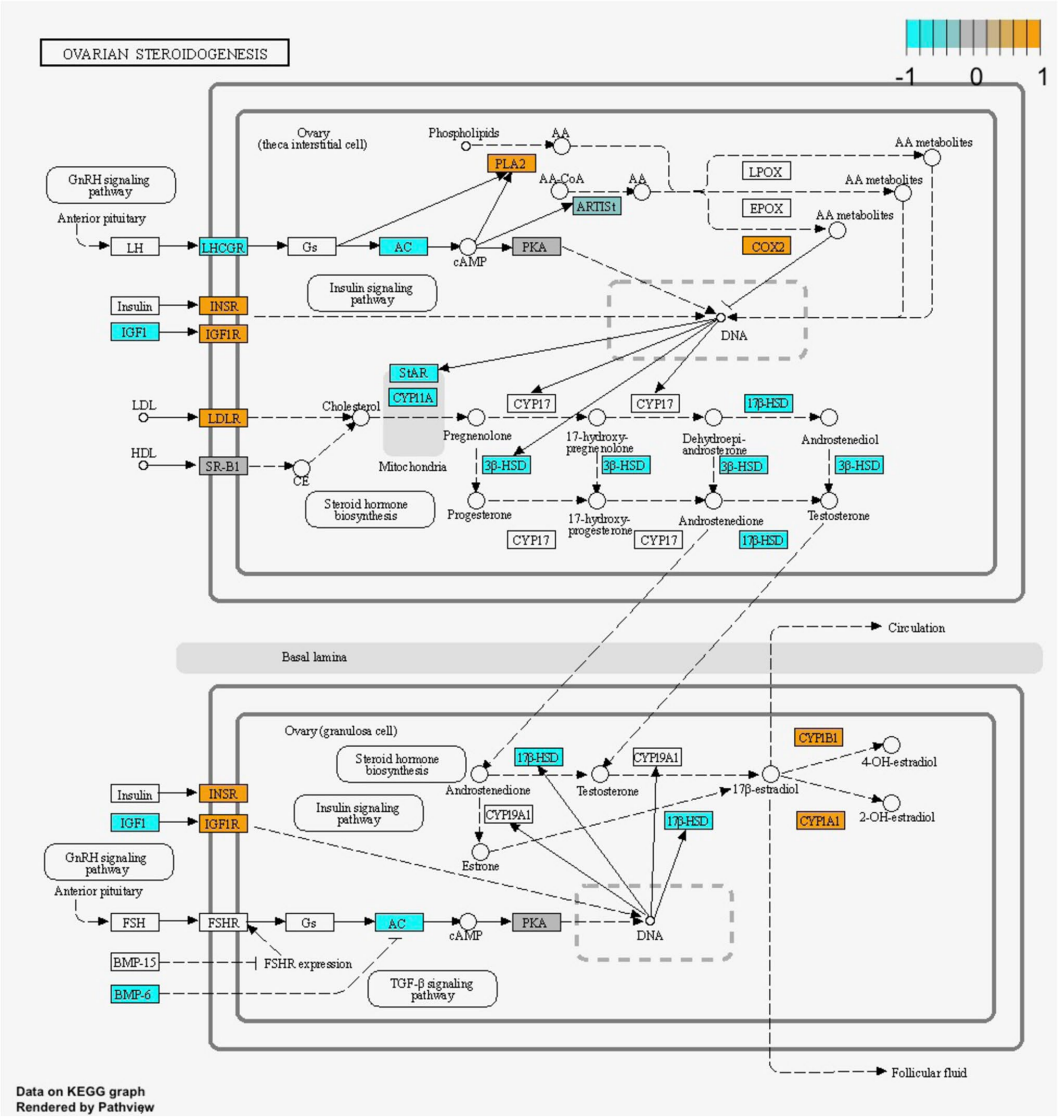
Alteration in ovarian steroidogenesis pathway during culture of vitrified tissue: Relative gene expression changes in the KEGG pathway illustrated in orange colour (upregulated gene expression during culture) and blue colour (downregulated gene expression during culture) for VTC vs. VT comparison. The upper panel represents relative gene expression changes in theca interstitial cells and the lower panel in granulosa cells. Undetected genes are shown in white. The grey colour indicates no change in expression

Due to their central involvement in ovarian follicle development and growth, we focused on WNT signalling and steroidogenesis pathways. The KEGG pathway for WNT signalling suggested changes in the canonical pathway and Ca +  + pathways with possible downstream impacts on cell cycle, cell adhesion and cytoskeleton (Fig. 8). Ovarian steroidogenesis and hormonal regulation were largely inhibited in the theca cells, with several key genes from *LHCGR* to *STAR*, *CYP11A1*, and *3b-HSD* being downregulated (Fig. 9). Given the importance of steroid production by growing follicles, and the interplay between WNT signalling and steroidogenesis, these pathways could be involved in the poorer follicle viability in vitrified tissue.

## Discussion

The current study aimed to provide a deeper understanding of the impact of vitrification on the ovarian cortex, assess the viability of the follicles after in vitro culturing, and search for data-driven ways to refine the existing vitrification protocols utilising bovine specimens as an animal model. To the best of our knowledge, this study is the first to conduct an in-depth examination between fresh and vitrified ovarian tissue and the subsequent in vitro culture of isolated follicles, along with the identification of (transcriptomic) pathways that could potentially improve tissue vitrification outcomes. This novel knowledge can contribute to making ovarian tissue vitrification a safer and more efficient approach for clinical use.

Cryopreservation and thawing of ovarian tissue pose significant challenges due to the presence of various cell types, including oocytes, follicular cells encapsulating oocytes and ovarian stromal cells that create a nourishing niche for developing follicles. Each one of these cell types exhibits individual susceptibility to freezing-associated stress factors that can result in cell damage. The extent of this cell damage could be linked to the freezing protocol applied. Previous research has shown that vitrification protocols are better at preserving ovarian stromal cells compared to slow freezing protocol [49, 50]. However, no significant differences in follicular viability have been observed among fresh, vitrified, and slow-frozen ovarian samples [50]. Despite these findings, the precise impact of vitrification protocols on follicles has remained largely unknown.

Furthermore, as previously reported, the vitrification procedure has several advantages. It can be learned faster compared to slow freezing, requires significantly less freezing time, and does not demand expensive equipment, resulting in reduced cost [51]. Additionally, the equipment for vitrification is lightweight and portable, enabling practitioners to perform the procedure in clinics near patients, thereby significantly enhancing the accessibility of fertility preservation treatments [52]. It is worth noting that despite of these advantages, most live births resulting from the use of cryopreserved ovarian tissue have been achieved through slow freezing protocols, while only four live births have been reported using the vitrification protocol [13, 14]. These outcomes reflect that vitrification, as a cost-effective cryopreservation technology, has been largely underexplored and clinically neglected.

The vitrification process relies on rapid cooling and the use of high concentrations of cryoprotectants to achieve a glassy, ice crystallisation free state, distinguishing it from the slow freezing method [53]. However, concerns arise due to the potential toxicity of the high concentration of cryoprotectants used in vitrification. As previously reviewed, several studies have attempted to vitrify ovarian tissue using cryoprotectants such as dimethyl sulfoxide (DMSO), propylene glycol (PrOH), EG or a combination of these [53]. Among these, EG stands out for its relatively low toxicity level and high cell permeability compared to other cryoprotectants [54]. In our study, we selected EG as the primary cryoprotectant to minimize toxicity. Apart from EG, the vitrification solution used in this study included sugar (sucrose) and polymer (Ficoll) as crucial components in vitrification media as they promote glass-like formation and reduce toxicity [55, 56]. Thus, we aimed to employ the least toxic, yet effective protocol for ovarian tissue vitrification.

The endpoint of a cryopreservation approach is to ensure a substantial population of follicles surviving the freezing and thawing process. Consequently, any potential harm to ovarian stroma and follicles may severely impact the recruitment and development of primordial follicles. In this study, the assessment of the vitrified ovarian tissue indicated no apparent differences between fresh and vitrified-thawed tissues (Fig. 2a; FT and VT). Similar findings have been reported by other studies using the same protocol [36, 57–59]. However, a severe impact of the culture on follicular morphology was revealed in FTC and VTC samples, with a noticeable increase in the density of atretic follicles (Fig. 2c) compared to non-cultured FT and VT tissue samples. Since only cultured tissue exhibited atretic follicles, it is plausible that this effect may be attributed to the stress induced by in vitro culture or the absence of oxygenated blood supply from the ovarian artery.

Importantly, the total viable follicle density remained unaffected in the FTC and VT groups when compared to FT (Fig. 2b). However, the assessment of follicle density in the VTC group revealed a significant decrease in monolayered and antral follicles, alongside a noticeable but insignificant trend towards an increase in secondary follicles (Fig. 2d). Previous research has suggested that cortical tissue fragmentation can trigger primordial follicle activation, leading to a marked decrease in the primordial follicle population and a trend towards an increase in the secondary follicle population [60]. This observation aligns with previous studies that have indicated that ovarian tissue fragmentation can disrupt Hippo signalling and stimulate Akt signalling, enhancing primordial follicle activation [61, 62]. Therefore, our findings on follicle density may reflect the consequences of excessive primordial follicle activation induced by the stress following vitrification, particularly after the additional 6 days of culture.

In this study, immunofluorescence for Ki67 revealed similar cell proliferation between FT and VT. However, an evaluation of cultured tissue disclosed a significant increase in cell proliferation (Fig. 3b) after comparing with the uncultured conditions, i.e., FTC vs. FT and VTC vs. VT. While this increase seems promising, it could also be a compensatory response to replace damaged or dead cells resulting from the suboptimal culture environment [63]. Notably the significant increase in the DNA-damaged cells observed in cultured tissue (Fig. 4b) confirmed the stress experienced by ovarian tissue during the in vitro culture. Additionally, previous research involving human ovarian tissue reported the association between cell proliferation and decreased follicle quality [64]. These findings suggest that the culture of ovarian tissue negatively impacts early follicle development.

Isolation and culturing of individual follicles were performed from fresh and vitrified-thawed ovarian tissue samples to assess their viability. Compared to the population of follicles isolated from fresh tissue, a reduced yield was obtained from the vitrified tissue, possibly due to cryodamage resulting from the vitrification procedure. However, H&E staining and the TUNEL assay performed on tissue samples showed no noticeable differences in follicle density (Fig. 2a) or DNA breaks (Fig. 4b) after vitrification. Initial studies on follicle isolation and viability indicated that the use of collagenase might affect follicle quality, potentially leading to basal lamina disruption [65]. The basal lamina plays a crucial role in cell growth and differentiation, providing mechanical support and essential signals [66]. Damage to the basal lamina during the isolation process could be detrimental to follicle viability. To minimise such damage, a previous study tested Liberase for follicle isolation, but this resulted in incomplete tissue disruption and failure to fully isolate the follicles [39]. Consequently, we employed a combination of low concentrations of collagenase IV, Liberase, and DNase I to obtain higher-quality isolated follicles. However, we cannot rule out the possibility that the vitrification process altered the extracellular matrix of ovarian tissue to a certain extent, which could have contributed to the reduced yield of isolated follicles following enzymatic treatment.

In this study, a total of 677 follicles were isolated, comprising 525 from fresh and 152 from vitrified tissue, respectively. After enzymatic isolation, the follicles exhibited a normal spherical shape and complete layers of granulosa cells, indicating minimal damage during the isolation process. Viability assessment immediately after isolation showed 93.5% and 87.5% live follicles from fresh and vitrified tissue, respectively (Fig. 5d, e). The viability assessment of the isolated follicles from the fresh tissue was consistent with previous studies [35, 67–69]. Moreover, the viability of the isolated follicles after vitrification/thawing in our study was comparable to that of follicles isolated from slow-frozen human ovarian tissue (82% viability of isolated follicles) [70]. In summary, our chosen follicle isolation protocol successfully provided high-viability isolated follicles for both fresh and vitrified tissue, essential for their further successful development.

The viability of the isolated follicles was evaluated every 2 days during the 6 days of in vitro culture. Isolated follicles were cultured in a group of 4–7 follicles embedded into an alginate bead [26], to determine whether the vitrification procedure affects their viability. Viability assessment of follicles isolated from both FT and VT revealed a similar trend in the distribution of viable follicles during their culture period (Fig. 5d, e). We did not observe a significant difference in the viability of the follicles for days 2 and 4 cultures (Fig. 5d, e). However, on day 6, we observed decreased survival of follicles in both fresh and vitrified tissue samples, with a more pronounced decline in the vitrified-thawed samples. The reduced viability observed in the follicles isolated from vitrified tissue following 6 day culture (23.1%) contradicted the findings of a previous study on slow-frozen human ovarian tissue, where 90% of isolated follicles remained alive after 7 days of culture [69]. Moreover, both from FT and VT we observed a well preserved 3D alginate structure, similar to previous study [67], suggesting that our culture system did not affect either the follicle structure or the microenvironment encapsulating the follicles. The dropped viability of the isolated and 6-day–cultured VT follicles also confirms our results from the histological analysis of ovarian tissue where we show a significant decrease in follicle density in VTC (Fig. 2b). Thus, further steps are required to improve the current protocol to obtain more comparable live follicles from both fresh and vitrified tissue.

To understand the vulnerable molecular pathways affected by tissue vitrification, we utilised bulk RNA sequencing for fresh and vitrified tissue samples with and without culture. From the analysis, we identified 175 DEGs in VT compared to FT, 158 DEGs in VTC compared to FTC, 1042 DEGs in FTC compared to FT and 1181 DEGs in VTC compared to VT. KEGG enrichment analysis of the DEGs was performed to observe the pathways that appear only in the vitrified groups. The DEG gene-sets between the vitrified and fresh samples, i.e., VT vs. FT and VTC vs. FTC, were not sufficient to reveal relevant KEGG terms, indicating negligible impact of vitrification on ovarian tissue transcriptomic activity.

Next, we tried to identify similar DEGs in our study to those from similar previous studies on transcriptomic profiles of vitrified ovarian tissue. Vitrification of ovarian tissue from domestic cats revealed 165 DEGs in vitrified compared to fresh tissue, involving the upregulation of mitochondrial genes related to respiration [71], which could not be identified in our study (Supplementary Table S1). Moreover, the vitrification of mouse ovaries using EG and DMSO as cryoprotectants revealed 623 DEGs [72]. However, only 7 DEGs overlapped with our DEG list. In addition, in a recent study using human ovarian vitrification, the authors identified 452 DEGs [73]; however, no overlap with our study was observed. These disparities might reflect variation in transcriptomic effects caused by vitrification, or differing technical aspects related to RNA-sequencing and down-stream data analysis. It is likely that all, the study design, the vitrification protocol used, ovarian tissue source as well as choice of transcriptomic platforms affect the results. However, these past studies together with our findings reveal a relatively minor impact of vitrification on genome activity of ovarian tissue.

The GSEA of the comparisons of cultured and uncultured tissue samples, i.e., FTC vs. FT and VTC vs. VT, reveals a series of significant common and unique DEGs and pathways. As shown from the Venn diagram, these comparisons share 725 DEGs (Fig. 6c). Thus, the pathways enriched with the unshared DEGs exist due to only the tissue vitrification before culture. Importantly, a significant difference was observed in the WNT pathway (Figs. 7 and 8 and Supplementary Table S3) enriched with DEGs in the vitrified groups demonstrating opposite directions of gene expression in the fresh tissue samples. WNT pathway secreted glycoproteins have an essential role in cell proliferation, migration, and fate [74]. Notably, the downregulation of *WNT11* and upregulation of *WNT5A* (Supplementary Table S3) were observed in the VTC group, while being opposite in fresh tissue samples, which may be affected by the vitrification process. Both *Wnt11* and *Wnt5a* expression have been reported in mouse GCs [75]; however, little is known on their function in the ovary. In humans, *WNT5A* is expressed in the luteinized GCs [76, 77], while in bovine GCs, *WNT5A* was found to have a negative impact by FSH [78], suggesting its role in follicle development and gonadotropin response [79]. Considering the downregulation of *WNT5A* observed in VT vs. FT (Fig. 8 and Supplementary Table S3), this might be one of the reasons for altered folliculogenesis after culturing (Fig. 2b). Moreover, the upregulation of *WNT5A* after culturing of ovarian tissue, as observed in our study for VTC, might be the reason for the reduced response to FSH and subsequently the lower success rates of live births reported when using vitrified tissue for autotransplantation [14].

We also observed up- and downregulation of *LHCGR* in VT and VTC, compared to FT and VT tissue samples, respectively (Supplementary Table S4). *LHCGR* is expressed in theca cells and is responsible for progesterone, androstenedione, and testosterone production, and is necessary for ovulation, luteinisation, and corpus luteum formation [80]. In our study, drop and increase in density of primordial/primary and secondary follicles in VTC, respectively (Fig. 2d) were linked with up- and downregulation of *LHCGR* in VT and VTC, respectively. This outcome is explainable by the assumption that vitrification of ovarian tissue upregulates *LHCGR*, which expression is downregulated during in vitro culture, causing more vigorous follicle recruitment in VT, but resulting in a lower density of antral follicle in VTC (Fig. 2d). Moreover, an increase in *GSK3β* (Fig. 8) in VTC samples may result in a decrease in β-catenin, as reported in a previous mouse study [81]. It has been reported that β-catenin promotes the expression of *LHCGR* [82]. Despite the fact that no differential expression was observed for β-catenin, both *GSK3β* and *LHCGR* differential expression support our hypothesis that upregulation of *GSK3β* contributes to the suppression of *LHCGR* in VTC samples (Fig. 8 and 9 and Supplementary Table S3 and S4). To conclude, the RNA sequencing results suggest that WNT signalling and ovarian hormonal regulation are the most vulnerable molecular pathways following vitrifying and thawing the ovarian tissue.

The current study broadened our understanding of how vitrification/thawing and culturing impact ovarian tissue and follicles, shedding light on the possible vitrification-related changes in follicular morphology, viability of isolated follicles, and tissue transcriptome. However, it is worth noting that a comparison with the slow freezing protocol was not included, which is a limitation that should be taken into consideration. This comparison could have provided valuable insights into the impact of slow freezing and vitrification on ovarian tissue, follicles, and transcriptomic profiles of the tissue samples.

It is also important to acknowledge that ovarian tissue heterogeneity is a possible limitation of our approach, as different pieces of ovarian tissue may have yielded different outcomes. However, we partially addressed this limitation by utilising a sufficiently large number of animals, ovaries and tissue pieces. Moreover, numerous studies have employed different approaches concerning the size of the tissue pieces used in experiments, primarily varying in length (ranging from 2.5 to 10 mm) and width (from 1 to 10 mm), while, maintaining a thickness of less than 2 mm [16, 17, 52, 83–86]. In our study, we followed these recommendations and used the tissue pieces of 2 × 5 mm in size with 1–1.5 mm in thickness. The choice of tissue sample size could influence the results, as larger samples may better preserve ovarian strips over time, which is essential for long-term graft function and follicle survival. Previous studies have also indicated that smaller grafts can lead to increased primordial follicle activation upon transplantation, depleting more quickly the follicles and reducing the lifespan of grafts [87, 88].

Utilising bovine ovarian tissue as a model for investigating vitrification techniques has produced diverse outcomes, with some studies supporting the method while others debate its shortcomings [17, 89, 90]. Therefore, additional research efforts are crucial to navigate the diversity of vitrification protocols and establish a standardised and effective approach for cryopreserving human ovarian tissue. Furthermore, beyond its application in human ovarian tissue cryopreservation, this method holds promise for animal breeding practices. Developing techniques using frozen ovarian tissue for culturing tissue pieces and isolating follicles to support their growth into mature oocytes could have significant benefits for animal breeding, the preservation of endangered species, the establishment of oocyte banks, and the enhancement of in vitro fertilisation procedures and fertility treatments for both animals and humans [91].

In conclusion, this study focuses on the effectiveness of ovarian tissue vitrification protocol using ethylene glycol as a cryoprotectant. It outlines shortcomings and possible directions for further studies before adapting the protocol for clinical use. The vitrification protocol used demonstrated minor damage to ovarian tissue, as evidenced by similar follicle density, and the overall tissue morphology, proliferation of cells, DNA double-strand breaks and the ovarian tissue RNA profile when compared to non-vitrified samples. However, during the culture of ovarian cortical tissue, a notable decrease in the total number of viable follicles was observed in vitrified-cultured tissue. Specifically, monolayered and antral follicles in the vitrified-cultured group showed a reduced number of follicles. The same results were confirmed by the viability analysis of the isolated follicles, where the vitrified group exhibited a significant drop in viability after 6 days of culture, unlike the non-vitrified group.

To provide data-driven cues for the future refinement the vitrification protocol, full transcriptome sequencing was employed. While the transcriptomic analysis did not reveal major effects of vitrification, changes were observed in WNT signalling and hormonal regulation when comparing fresh-cultured and vitrified-thawed and cultured tissue samples. Differential expression of WNT and hormonal regulation pathway genes in vitrified and cultured ovarian tissue indicates to them as vulnerable molecular pathways and potential targets for the vitrification protocol refinement. As a result of this, future studies could focus on the appending of the vitrification or thawing media by adding LH and/or WNT modulators, in order to overpass the vulnerable molecular pathways observed in our study. In summary, this study indicates that vitrified ovarian tissue behaves similarly to fresh ovarian tissue, with only minor changes observed. These findings suggest the potential ways for optimising vitrification protocol for ovarian tissue preservation before it can be clinically adapted.

### Supplementary Information

Below is the link to the electronic supplementary material.

Supplementary file1 (XLSX 338 KB)

Supplementary file2 (XLSX 29 KB)

Supplementary file3 (XLSX 24 KB)

Supplementary file4 (XLSX 19 KB)

## Data Availability

The data are available from the corresponding author upon request.
